# T-Cell Acute Lymphoblastic Leukemia/Lymphoma (T-ALL) With Negative Screening Immaturity Markers and Gamma-Delta Receptor Expression

**DOI:** 10.7759/cureus.57399

**Published:** 2024-04-01

**Authors:** Maria Faraz, Anita Parmigiani, Nina Monkash, Anne Chen

**Affiliations:** 1 Department of Pathology and Laboratory Medicine, Albany Medical College, Albany, USA; 2 Department of Radiology, Albany Medical Center, Albany, USA; 3 Department of Pathology & Immunology, Washington University in St. Louis, St. Louis, USA

**Keywords:** cd34 expression, immaturity markers, tdt, gamma delta, tcr, t-cell receptor, t-all

## Abstract

T-cell acute lymphoblastic leukemia/lymphoma (T-ALL) is characterized by the combination of T-cell lineage and the presence of immaturity marker(s). Sometimes, the most common immaturity markers for initial flow cytometry screening in T-ALL may be negative, which can be a diagnostic pitfall. When a lack of common first-line immaturity markers is encountered in combination with gamma/delta T-cell receptor expression, a misdiagnosis of mature gamma-delta T-cell leukemia/lymphoma could be rendered. Here, we discuss two T-ALL cases with the absence of common flow cytometry immaturity markers and positive gamma/delta receptor expression.

## Introduction

T-cell acute lymphoblastic leukemia/lymphoma (T-ALL) constitutes approximately 12-15% of acute lymphoblastic leukemia cases in the pediatric population [1,2]. The diagnosis of T-ALL hinges on key factors, including lineage determination, clonality assessment, and the presence of immature markers [3,4]. Among the essential immaturity markers, Terminal deoxynucleotidyl transferase (TdT) plays a pivotal role. As an intranuclear protein, TdT is expressed during the early stages of lymphopoiesis, serving as a valuable marker for identifying immature lymphoid cells. Notably, TdT is expressed in 90-95% of lymphoblastic lymphomas, and its absence poses challenges to the diagnosis of T-ALL [3,5,6].

T-cells undergo genotypic changes through T-cell receptor (TCR) rearrangement. T-lymphocytes exhibit two types of receptors: alpha-beta T cells, predominant in the majority of peripheral T lymphocytes, and gamma-delta receptors, present in a minority (approximately 1-5%) [7]. Notably, the majority of T-ALL have neither alpha-beta nor gamma-delta TCR expression, with gamma-delta expression seen in only 9% of all T-ALL [8].

In this context, we present two unique cases of T-ALL characterized by less pronounced immaturity marker positivity in initial flow cytometry screening and the presence of gamma-delta T-cell receptor positivity. These cases underscore the diagnostic complexities associated with T-ALL, particularly when immaturity markers exhibit atypical expression patterns and rare TCR expression is involved.

## Case presentation

Case 1

**Figure 1:**
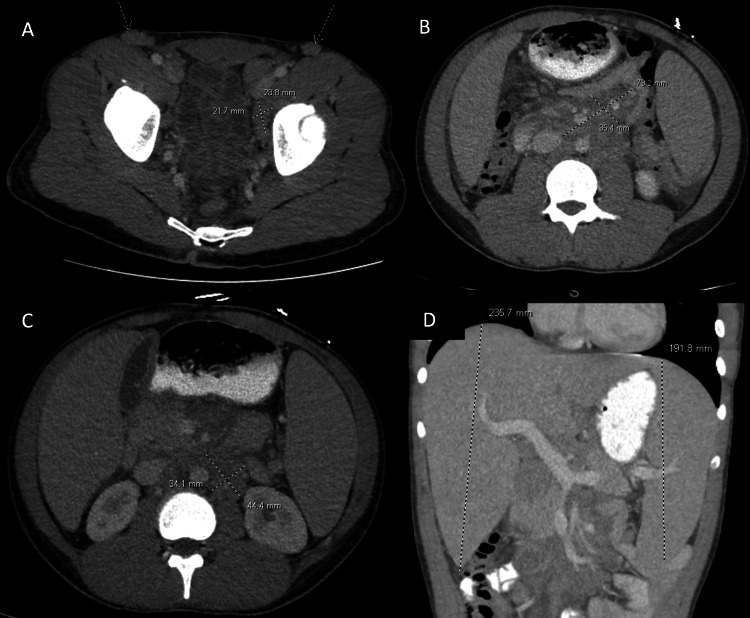
Case 1: computed tomography (CT) showing lymphadenopathy and hepatosplenomegaly Mesenteric and retroperitoneal lymphadenopathy. Axial and coronal intravenous contrast material–enhanced CT image shows (A) left external iliac nodal mass measuring 2.88 x 2.17 mm (dashed cross) and bilateral inguinal lymphadenopathy (dashed arrows). (B) A central mesenteric conglomerate nodal mass measuring 7.32 x 3.54 mm (dashed cross). (C) Left para-aortic nodal mass measuring 4.44 x 3.41 mm (dashed cross). (D) Hepatosplenomegaly (dashed lines).

An 18-year-old male with no significant medical history had a tooth extraction surgery and was diagnosed with incidental lymphadenopathy. Subsequently, he developed episodes of epistaxis and bruising, which gradually became more frequent. Initial CBC counts showed a white blood cell count of 33.8 x103/ µl with abnormal white blood cells differential count of 63%, hemoglobin of 10.4 g/dl, and platelets of 19,000 x 103/ µl. Upon referral to our institution, his computed tomography scan (Figure 1) showed mesenteric and retroperitoneal lymphadenopathy, as well as hepatosplenomegaly.

**Figure 2:**
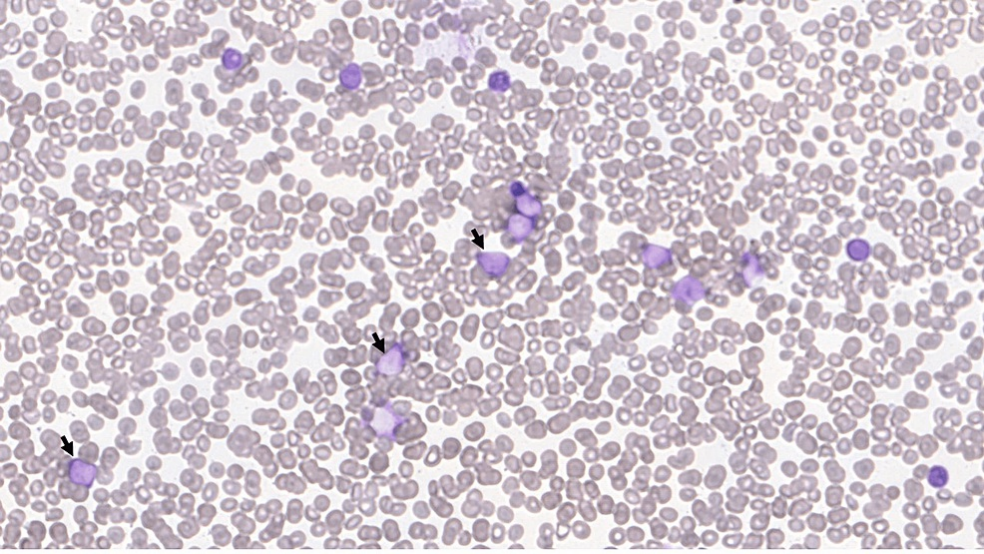
Case 1: peripheral blood smear, 40x, showing blast-like cells (arrows) While the majority of cells shown in this peripheral smear are likely neoplastic cells, the black arrows indicate cells that show the characteristic blast-like morphology.

Hepatosplenomegaly was also present. Peripheral blood smear (Figure 2) showed circulating blast-like cells.

**Figure 3:**
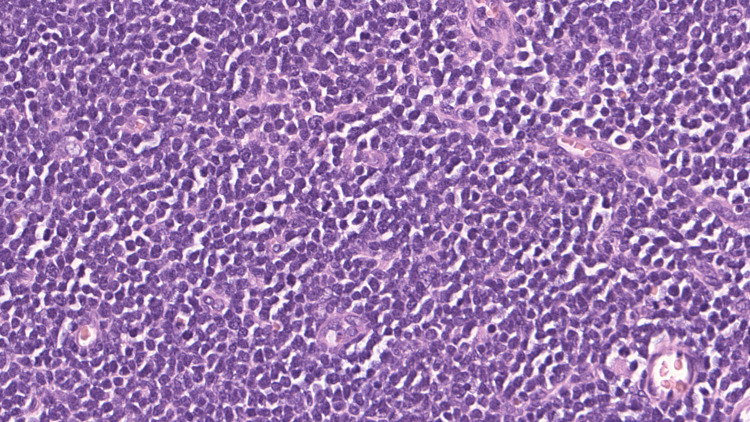
Case 1: Lymph node histology, hematoxylin & eosin, 40x, showing diffuse sheets of neoplastic cells

Lymph node biopsy and flow cytometry analyses were performed. The lymph node showed sheets of abnormal lymphoid cells that were small to intermediate in size with a high nuclear-cytoplasmic ratio, moderately dispersed, fine chromatin, round nuclear contours, and scant cytoplasm (Figure 3).

Immunohistochemistry

**Figure 4:**
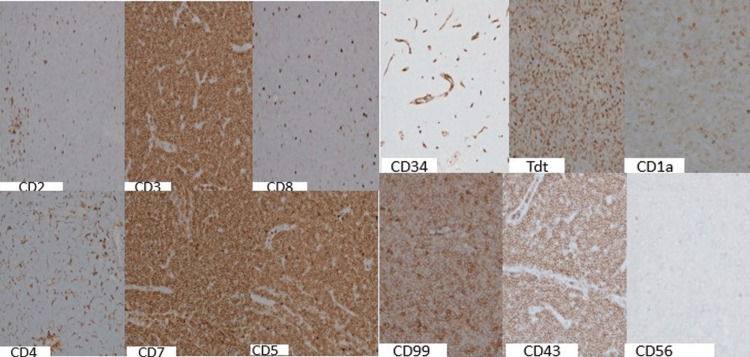
Case 1: immunohistochemical stain results, as labeled, 10x, showing that the abnormal cells express many T-cell markers as well as immaturity markers

The lymph node neoplastic cells were strongly and diffusely positive for pan-T-cell markers CD3, CD5, and CD7. Also, tumor cells showed positivity with T-cell markers CD2 (variable/subset) and CD8 (majority dim); immaturity markers CD99 (moderate to bright diffuse); TdT/Tdt (variable); and CD1a. CD34, CD56, and CD4 showed no significant expression. Immunohistochemistry results are shown in Figure 4.

Flow Cytometry

**Figure 5:**
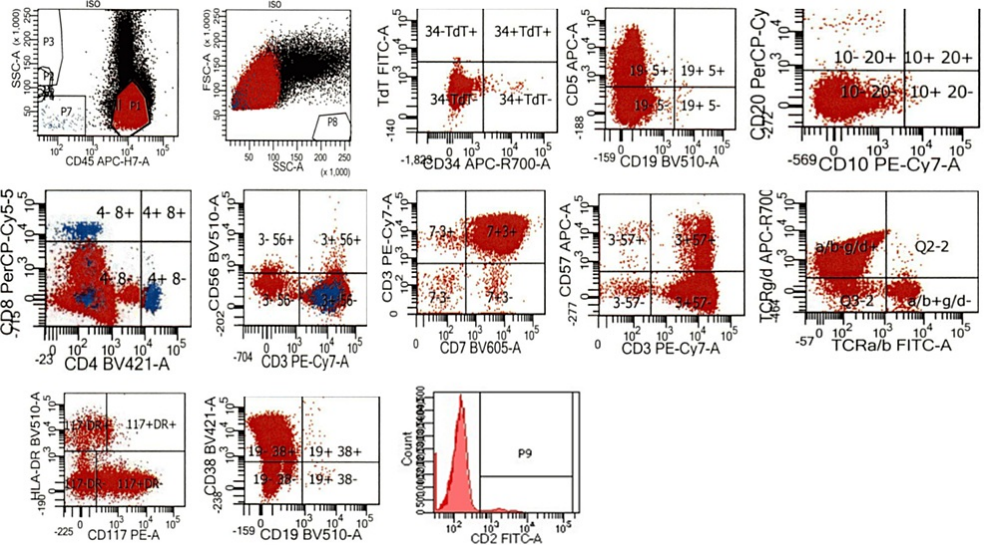
Case 1: flow cytometry plots characterizing antigen expression of the abnormal cell population The abnormal cell population is shown in red in all plots; normal elements in the specimen (monocytes, neutrophils) are shown in black. Dot plots in the first row show forward (FSC) and side (SSC) scatter properties of the abnormal cell population and its expression of CD45, CD34, TdT (nuclear staining), CD19, CD5, CD10, and CD20. Dot plots in the middle row show, residual normal T-cells highlighted in blue, expression of CD4, CD8, surface CD3, CD56, CD7, CD57, TCR alpha/beta (a/b), and TCR gamma/delta (g/d). Dot plots in the bottom row show the expression of CD117, HLA-DR, CD38, CD19, and CD2.

Multiparametric flow cytometry analysis (Figure 5) for peripheral blood and axillary lymph nodes detected 90% abnormal cells of T-cell lineage with positive expression for cytoplasmic and surface CD3, dim CD5, bright CD7, CD38, bright CD45, TCR gamma-delta, and partial dim CD117, and largely negative for CD2, CD8, CD10, CD26, CD34, HLA-DR, TdT and TCR alpha-beta.

Molecular Study

Deletion of CDKN2A, located on 9p21, was identified by a next-generation sequencing lymphoid panel.

Treatment and Outcome

The patient was treated per Children's Oncology Group Protocol AALL0434 [9]. The first post-treatment bone marrow contained a small amount of residual disease, with a similar marker profile as was present at diagnosis; this was detected by flow cytometry and this abnormal cell population was not definitively seen morphologically. He refused additional chemotherapy after a few cycles, relapsed, and passed away approximately one year after the initial diagnosis.

Case 2

**Figure 6:**
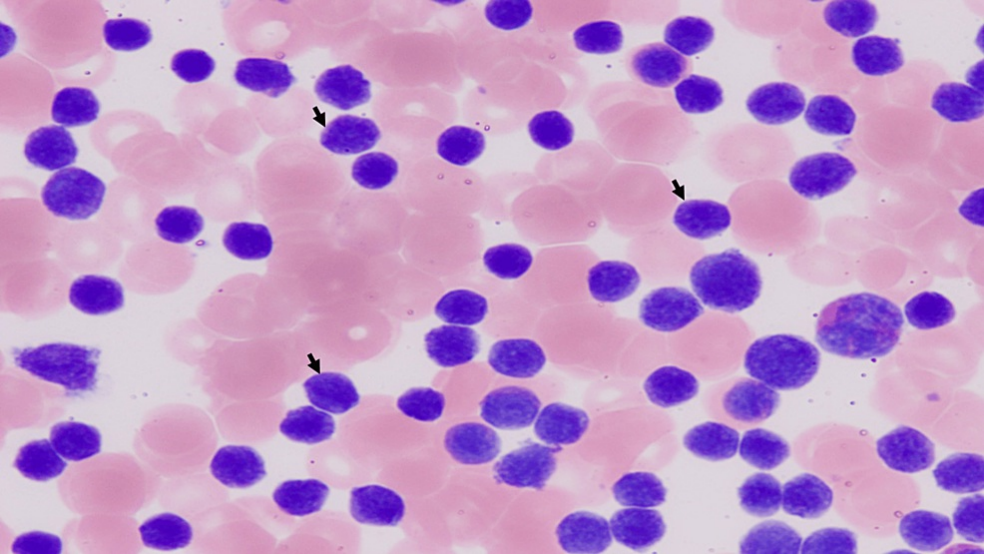
Case 2: peripheral blood smear, 100x, showing a monotonous, atypical cell population (arrows) While the majority of cells in this image likely are neoplastic cells, the black arrows indicate cells with the characteristic atypical morphology.

A 19-year-old female presented to the emergency room with a two-day history of nausea and vomiting, accompanied by a week-long loss of appetite and occasional diarrhea. Upon admission, her initial complete blood count (CBC) revealed a white cell count of 890,000 x 10^3/µl, with 92% abnormal white blood cells. The peripheral blood smear is shown in Figure 6.

**Figure 7:**
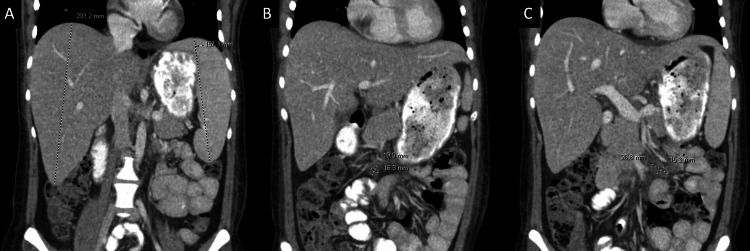
Case 2: computed tomography showing mesenteric adenopathy and hepatosplenomegaly (indicated) Moderate lymphadenopathy within the small bowel mesentery. Coronal intravenous contrast material–enhanced CT images show (A) hepatosplenomegaly (dashed lines) and (B and C) prominent mesenteric lymph nodes (dashed crosses) in the superior mesenteric artery/superior mesenteric vein mesenteric distribution.

Serum chemistry indicated hyperkalemia, and on physical examination, hepatosplenomegaly was noted. Abdominal computed tomography (Figure 7) revealed mild to moderate mesenteric lymphadenopathy and hepatosplenomegaly.

**Figure 8:**
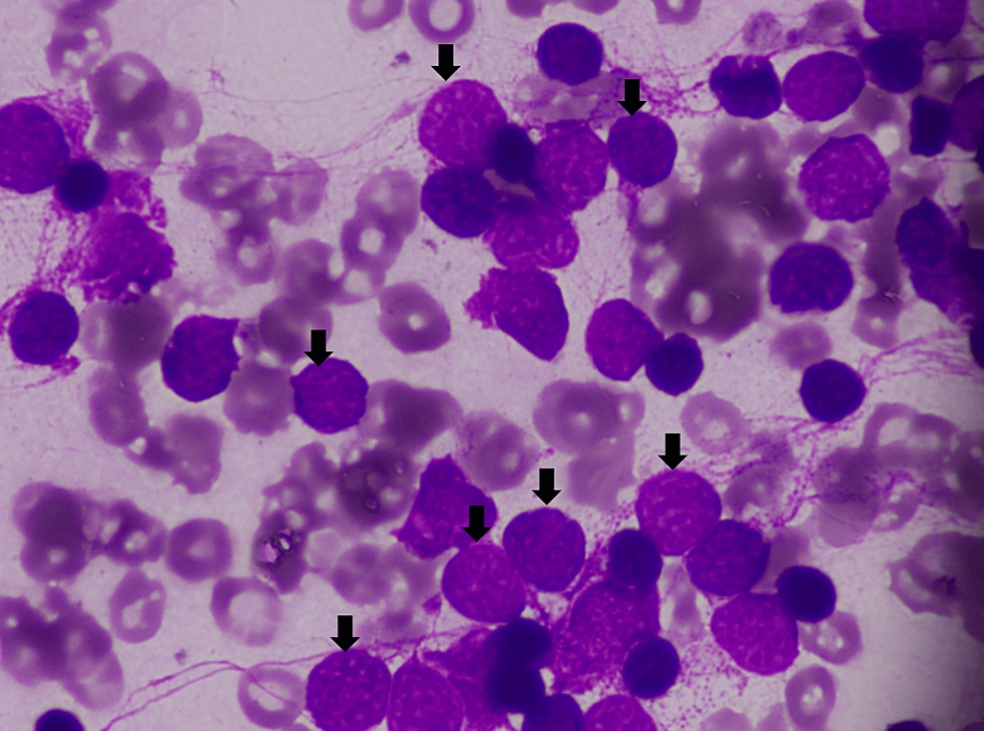
Case 2: bone marrow aspirate, 100x, showing atypical cells with somewhat immature-appearing chromatin (arrows) While the majority of cells in this image are likely neoplastic cells, the black arrows show cells with the characteristic atypical morphology.

Subsequently, a bone marrow aspiration was performed. The bone marrow aspirate (Figure 8) depicted numerous abnormal lymphocytes, characterized by small to intermediate cell size, high nuclear-cytoplasmic ratio, moderately dispersed and fine chromatin, round nuclear contours, and scant cytoplasm.

Flow Cytometry

**Figure 9:**
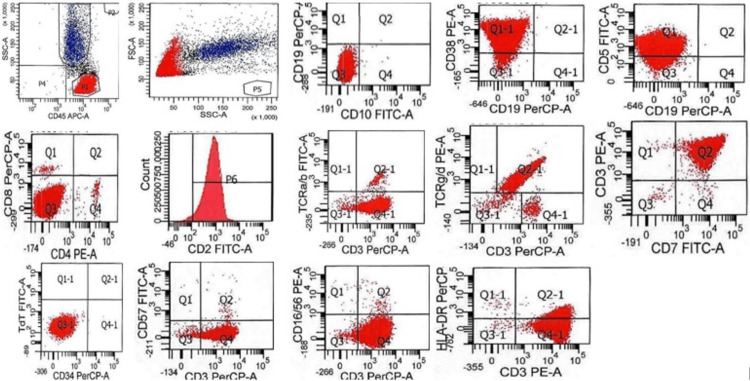
Case 2 Flow cytometry plots characterizing antigen expression of the abnormal cell population The abnormal cell population is shown in red in all plots; normal elements in the specimen (neutrophils) are shown in blue. Dot plots in the first row show forward (FSC) and side (SSC) scatter properties of the abnormal cell population and its expression of CD45, CD10, CD19, CD38, and CD5. Dot plots in the middle row show expression of CD4, CD8, CD2, surface CD3, TCR alpha/beta (a/b), TCR gamma/delta (g/d), and CD7. Dot plots in the bottom row show expression of CD34, TdT (nuclear staining), surface CD3, CD57, CD16/CD56, and HLA-DR.

Multiparametric flow cytometric analysis (Figure 9) demonstrated 91% abnormal cells of the T-lymphoid lineage. The cells exhibited positive expression for CD2, CD3, heterogenous CD5, CD7, TCR gamma/delta, CD38, and CD45. The cells were negative for TCR alpha/beta, CD4, CD8, CD16/CD56, CD10, CD34, and TdT.

Treatment and Outcome

The patient received induction therapy with a Children's Oncology Group Protocol consisting of dexamethasone, vincristine, doxorubicin, and PEG asparaginase, with no remission. She was then treated with four doses of moderate-dose cytarabine, with no remission. She was then treated with nelarabine, etoposide, and cytarabine, with no remission. She then received high-dose cytarabine plus mitoxantrone and again, failed to respond. She finally achieved remission with alemtuzumab; however, this was complicated by CMV reactivation. At this point, she underwent an allogenic stem cell transplant with no initial complications. The patient relapsed at approximately three months post-transplant and was treated with ribociclib with no significant response. She ultimately passed away approximately one year after the initial diagnosis. 

## Discussion

The diagnosis of lymphoid neoplasms relies on a comprehensive evaluation of morphologic, immunophenotypic, and genetic information. The World Health Organization (WHO) classification categorizes lymphoid neoplasms into B-cell or T-cell lineages, further distinguishing between mature and immature neoplasms based on cell characteristics [10,11]. While, by morphology alone, T-ALL is not definitely distinct from B-ALL, the immune profile becomes instrumental in its identification. CD3, CD7, and CD5 are crucial markers indicative of T-cell lineage, with CD3 being the most lineage-defining for T-cells.

Both of our cases exhibited positivity for pan-T-cell markers, in particular, the lineage-defining CD3. There were various T-cell aberrancies, including bright CD7 and dim CD5 and double-negativity for CD4 and CD8, suggesting T-cell clonality. Subsequently, the focus shifted to determining the maturity of these abnormal T cells. Widely available markers to support T-cell immaturity include TdT, CD34, CD1a, CD99, HLA-DR, and CD117 [12]. Although both cases were largely negative for CD34, HLA-DR, and TdT on flow cytometry, the immunohistochemical staining profile of the first case showed variably positive TdT immunostaining, and had bright CD1a and CD99 positivity, contributing to the diagnosis of T-ALL. Immunohistochemical staining was not available for Case 2.

Importantly, the absence of TdT does not entirely exclude the possibility of T-ALL, as evidenced by existing literature reporting TdT-negative cases [13]. There are several reports in the literature of TdT negative T-ALL: Faber et al. published 3 out of 200 cases of T-All with negative TdT [5]; Suzumiya et al. in their study reported TdT positivity in 94% of cases [14]; and Gupta et al. studied 38 cases and reported TdT positivity in 39.5% cases, and CD34 positivity in 34.2% cases [3].

Regarding T-cell receptor expression, T-ALL typically exhibits absent TCR expression, with only 9% of T-ALL showing gamma-delta rearrangements [8,15]. Interestingly, both cases in our study demonstrated gamma-delta rearrangements, and the first case further exhibited CDKN2A/B deletion, reinforcing the T-ALL diagnosis [16].

The uniqueness of our cases lies in their absence of the common screening immaturity markers, CD34 and TdT, and the rare expression of gamma/delta T-cell receptors. Differential diagnoses, such as mature T-cell neoplasms and NK-ALL, were considered but ruled out based on the brighter CD7 and CD5 expression and the absence of CD57, CD56, and CD16 [3,12]. Early T-cell precursor neoplasm was also considered for the first case, but features such as partial dim CD117, positive expression of CD5 and CD3 on T-cells, and reported CD117 positivity in 11.2% of T-ALL cases helped exclude this possibility [15,17]. While these features provided valuable insights, no single feature is diagnostic of these entities. Therefore, the combination of morphological, immunophenotypic, and genetic analyses in our cases is most consistent with T-ALL with gamma/delta differentiation.

## Conclusions

In particular, these two cases in our series had short survival intervals of only approximately one year. The absence of common immaturity markers by a limited initial flow cytometry panel should not on its own exclude immaturity and T-ALL. The immaturity of the neoplasm in these two cases was confirmed based on H&E appearance and immunohistochemical stains CD1a and CD99, which were not available in our limited screening flow cytometry panel. The correlation of clinical presentation with morphological, immunophenotypic, and genetic findings is essential for an accurate diagnosis.
